# The Status of Metabolic Control in Patients With Type 2 Diabetes Attending Dasman Diabetes Institute, Kuwait

**DOI:** 10.3389/fendo.2019.00412

**Published:** 2019-06-26

**Authors:** Mohammad Qaddoumi, Yousuf Al-Khamis, Arshad Channanath, Jaakko Tuomilehto, Dalia Badawi

**Affiliations:** ^1^Dasman Diabetes Institute, Kuwait City, Kuwait; ^2^Faculty of Pharmacy, Kuwait University, Kuwait City, Kuwait; ^3^Center for Vascular Prevention, Danube-University Krems, Krems an der Donau, Austria; ^4^Department of Chronic Disease Prevention, National Institute for Health and Welfare, Helsinki, Finland

**Keywords:** type 2 diabetes, glycemic control, HbA1c, anti-diabetic medication, diabetic complications

## Abstract

**Purpose:** To evaluate metabolic control in patients with type 2 diabetes at Dasman Diabetes Institute (DDI, Kuwait), a specialist diabetes clinic and research center, and to investigate its association with patient demographics and clinical characteristics.

**Methods:** Data from 963 patients with type 2 diabetes were retrospectively collected from the Knowledge Based Health Records maintained at DDI for patients who attended DDI during 2011–2014. The collected data included patient demographics, clinical characteristics, and anti-diabetic medications. Student's *t*-test was used to evaluate the differences in mean values between poor and good glycemic control groups. Categorical variables were assessed using chi-square analysis with Fisher's exact test for small data sets.

**Results:** The patients' mean age was 53.0 ± 9.5 years with equal number of males and females. Females (34.4 ± 7.2 kg/m^2^) had a higher mean body mass index than males (32.1 ± 6.4 kg/m^2^). The mean fasting blood glucose and HbA1c levels were 9.6 ± 3.8 mmol/L and 8.5 ± 1.8%, respectively. Dyslipidemia (46%) and hypertension (40%) were the most common comorbidities, whereas nephropathy (36%) and neuropathy (35%) were the most common diabetic complications. The most commonly used anti-diabetic medication was metformin (55%). Factors significantly associated with poor glycemic control (HbA1c level ≥ 7%) included insulin use; neuropathy or foot ulcers as diabetic complications; and elevated systolic blood pressure and total cholesterol, low-density lipoprotein (LDL) cholesterol, triglycerides, and fasting blood glucose levels. Factors significantly associated with good glycemic control included metformin use and elevated high-density lipoprotein cholesterol level. The proportion of patients with good glycemic control (HbA1c level < 7%) was 29.5%. A large proportion of the patients with poor glycemic control were only administered monotherapy drugs, and two-thirds of the patients were obese. Further, the American Diabetes Association (ADA) recommendations for blood pressure and LDL cholesterol level were met (62 and 63%, respectively) by follow-up year 4.

**Conclusion:** The therapeutic management of type 2 diabetes in Kuwait is suboptimal. Therapeutic strategies should ensure better adherence to ADA guidelines, evaluate the high obesity rates, and adherence to lifestyle recommendations by patients, and continually promote diabetes education and self-empowerment.

## Introduction

Diabetes mellitus is a chronic metabolic disease that is known to have affected 415 million people worldwide in 2015. It has been projected that >600 million people will acquire the disease by 2040 (1). Kuwait is one of the countries with the highest prevalence of diabetes mellitus globally, with an estimated prevalence of 25.4% reported among adults in 2013 (2). The recent increase in the prevalence from 14.8% in 1998 in Kuwait is alarming (3). Generally, 90–95% of patients with diabetes are classified as having type 2 diabetes characterized by the lack of response to the effects of insulin by the human body or its inability to produce enough insulin (4).

Patients with diabetes are prone to the development of microvascular complications, such as nephropathy, neuropathy, and retinopathy, and macrovascular complications, such as coronary artery disease, stroke, and heart failure. These diabetic complications result in marked disability, mortality, and an enormous national economic burden if not managed well (5). One way of reducing diabetic complications associated with type 2 diabetes and improving its long-term outcome is ensuring tight control of blood glucose level and blood pressure. For instance, the UK Prospective Diabetes Study (UKPDS) demonstrated that intensive glycemic control with anti-diabetic medications is vital for preventing the chronic complications associated with type 2 diabetes. Furthermore, tight blood pressure control in patients with hypertension and type 2 diabetes reduced the risk of death related to diabetes and its complications and reduced the progression of diabetic retinopathy and blindness (6, 7).

According to the American Diabetes Association (ADA), a glycated hemoglobin (HbA1c) level of <7% indicates good glycemic control in patients with diabetes. It has been shown that an improvement in HbA1c level by 1% in patients with type 2 diabetes reduced the risk of microvascular complications by 37% and those of heart failure and myocardial infarction by 16 and 14%, respectively (8).

However, attainment of glycemic control by patients with diabetes has not been adequate worldwide. The cross-sectional PANORAMA study that analyzed the data for adults with type 2 diabetes from nine European countries showed that only 37.4% of these patients achieved the target HbA1c level of <7% (9). Furthermore, a large prospective study that observed a combination of diabetic patients from 141 study centers located in the Czech Republic and Slovakia determined that only 29.9% of patients with type 1 diabetes and 33.4% of those with type 2 diabetes attained the desired target HbA1c level of <7% (10). Similarly, a local study that collected data from patients with type 2 diabetes from 28 health centers in Saudi Arabia showed that only 27% of these patients reached the target HbA1c level of <7% (11). Hence, the purpose of this retrospective study was to evaluate the level of metabolic control in patients with type 2 diabetes at a specialist diabetes clinic and research center located in Kuwait and to investigate associations between glycemic control and patient demographics, clinical characteristics, and anti-diabetic medications.

## Methods

### Study Population

In this study, data from a total sample of 1,191 patients with type 2 diabetes were obtained from the Knowledge Based Health Records (KBHR), an electronic health record system maintained at Dasman Diabetes Institute (DDI), a specialist diabetes clinic and research center in Kuwait. The inclusion criteria were type 2 diabetes patients (excluding pregnant patients) aged 18–70 years who attended the clinics at DDI from 2011 to 2014 and were enrolled at DDI for ≥1 year prior to this period, had at least three endocrinologist appointments per year, and had their HbA1c levels measured at least twice annually. Based on the inclusion criteria, the total sample was 963 patients with type 2 diabetes. The collected data included patient demographics, clinical characteristics, anti-diabetic medications dispensed by the pharmacy, and laboratory results such as measured serum creatinine, lipid profile, glycated hemoglobin level, and fasting blood glucose level.

The included patients were stratified into categories according to their last recorded HbA1c level and/or prescription patterns taken in their last appointment for each year. Good glycemic control was defined as HbA1c <7% and poor glycemic control as HbA1c ≥7%. The outcomes of interest were evaluated by examining the patients' latest laboratory results. Annual adherence of patients to performance indicators was evaluated every 12 months, and values were placed in 12 month block intervals using their measurements obtained at the initial visit as the reference starting point. Prescription patterns were classified into three main categories: monotherapy, combination therapy, and total therapy (sum of monotherapy and combination therapy). Prescription pattern was defined as the number of anti-diabetic medications prescribed in the latest prescription. For example, if a patient was started with metformin but later switched to a sulfonylurea, his/her treatment was categorized into a sulfonylurea monotherapy. If a patient started with metformin and later a sulfonylurea was introduced, then the patient was categorized into a combination therapy. Six indicators were adopted to measure the performance in relation to diabetes management, three of which were process indicators and three were outcome indicators.

### Process Indicators

The following process indicators were used: glycosylated hemoglobin (1) HbA1c management, percentage of patients who underwent ≥1 HbA1c tests annually; (2) cholesterol/lipid measurement, percentage of patients who underwent ≥1 low-density lipoprotein (LDL) cholesterol test annually; and (3) annual screening of nephropathy, percentage of patients who underwent ≥1 test for urinary microalbumin level measurement during the measurement year. The urinary microalbumin test is a urine test that measures the amount of albumin in the urine. When kidney damage occurs, albumin leaks into the bloodstream and it is present in urine.

### Outcome Indicators

The following outcome indicators were used: (1) HbA1c control, percentage of patients with the most recent HbA1c level of <7%; (2) LDL cholesterol control, percentage of patients with the most recent LDL level of <2.6 mmol/L; and (3) blood pressure control, percentage of patients with the most recent blood pressure level of <140/90 mmHg.

### Statistical Analysis

Results are expressed as mean ± SD or frequencies (and proportions). Student's *t*-test was performed to evaluate the differences in mean values between the poor and good glycemic control groups. Categorical variables were assessed by performing chi-square analysis with Fisher's exact test when the number of data points was small. For differences among variables, a *p* < 0.05 was considered statistically significant. All analyses were performed using R version 3.5.1: A language and environment for statistical computing.

### Ethical Approval

The study obtained ethical approval from the Ethical Review Committee at DDI in 2014 to conduct it and to permit access to the patient data from the KBHR electronic health record database. All patients attending DDI signed a consent form, which allowed their information to be used for any research purpose. To maintain privacy and anonymity, all patient data were extracted without identifying name, address, or national ID number and a unique identification was assigned to each participant. The patient data will be kept confidential by the study investigators, and all paper and electronic records of the patients will be stored securely and limited only to authorized study investigators.

## Results

### Population Characteristics

Out of a total of 1,191 patients with type 2 diabetes, 963 (81%) patients met the inclusion criteria and their detailed demographic and clinical data were collected. The demographics and clinical characteristics of the patients with type 2 diabetes at baseline are presented in Table 1. Among these 963 patients, the number of females and males was similar. The overall mean age of the cohort was 53.0 ± 9.5 years. The mean body mass index (BMI) was higher in female (34.3 ± 7.0 kg/m^2^) than in male patients (32.1 ± 6.1 kg/m^2^). The mean levels of total cholesterol, LDL cholesterol, high-density lipoprotein (HDL) cholesterol, and triglycerides were 4.7 ± 1.1, 2.8 ± 0.9, 1.1 ± 0.3, and 1.8 ± 1.4 mmol/L, respectively. Further, the mean fasting blood glucose and HbA1c levels were 9.6 ± 3.8 mmol/L and 8.5 ± 1.8%, respectively. Among all comorbidities, dyslipidemia (46.5%) and hypertension (40.4%) were the most common in the study population, whereas the most common diabetic complications were nephropathy (36.7%) and neuropathy (35.4%) followed by retinopathy (21.7%).

**Table 1:** Demographic and clinical characteristics of T2D patients (n = 963).

**Variable**	**Number (%)**
**Age**	
mean ± SD (years)	53.0 ± 9.5
<50	274 (28.5)
50–65	656 (68.1)
>65	33 (3.4)
**Sex**	
Male	483 (50.0)
Female	480 (49.8)
**BMI, Mean ± SD (KG/M**^**2**^**)**	
Male	32.1 ± 6.1
Female	34.3 ± 7.0
SBP, mean ± SD (mm/Hg)	134.4 ± 17.7
DBP, mean ± SD (mm/Hg)	75.8 ± 11.3
Total cholesterol, mean ± SD (mmol/L)	4.7 ± 1.1
LDL cholesterol, mean ± SD (mmol/L)	2.8 ± 0.9
HDL cholesterol, mean ± SD (mmol/L)	1.1 ± 0.3
Triglyceride, mean ± SD (mmol/L)	1.8 ± 1.4
Fasting blood sugar, mean ± SD (mmol/L)	9.6 ± 3.8
HbA1c level, mean ± SD (%)	8.5 ± 1.8
Creatinine, mean ± SD (μmol/L)	85.3 ± 32.8
***Comorbidities and diabetic complications***	
Dyslipidemia	449 (46.52)
Hypertension	391 (40.4)
Nephropathy	353 (36.7)
Neuropathy	341 (35.4)
Retinopathy	209 (21.7)
Coronary heart disease	95 (9.9)
Foot ulcer	54 (5.6)
Stroke	15 (1.6)
Kuwaiti	720 (74.8%)
Average diabetes appointment per year	2.3

*Mean values of LDL, HDL, HbA1c, and BP levels are calculated on the basis of the values measured at baseline of the study patients*.

Table 2 summarizes the characteristics of the medicines administered to the study patients. As shown, the majority of the patients received monotherapy with an oral drug without insulin. The most common anti-diabetic medication administered as monotherapy was metformin (21.0%) followed by insulin (20.3%) and DPP-4 inhibitors (8.3%), with glitazones (mainly pioglitazone) being the monotherapy medication administered to the least number of patients (0.2%). Regarding combination treatment, most patients received one oral drug with insulin (12.1%) followed by two oral drugs without insulin (11.3%). The most common combination treatment was metformin with either insulin (9.8%) or a DPP-4 inhibitor (7%) or both (6.3%). The least used drug combination was a sulfonylurea with insulin and a DPP-4 inhibitor (0.1%). Further, only 3.1% of the patients received no anti-diabetic therapy and were managed on diet and/or with lifestyle changes. The most common anti-diabetic medication prescribed in total (as monotherapy or combination treatment) was metformin (55.2%) followed by insulin (42.9%).

**Table 2:** Medicine characteristics of T2D patients.

**Anti-diabetic medication**	**Number (%) of patients receiving the therapy**
**MONOTHERAPY**	
Metformin	202 (21.0)
Insulin	196 (20.3)
DPP-IV inhibitors	80 (8.3)
Sulfonylureas	63 (6.5)
GLP-1 agonists	16 (1.6)
Meglitinides	10 (1.0)
Glitazones	2 (0.2)
1 oral drug without insulin	373 (38.7)
**COMBINATION THERAPY**	
Metformin + insulin	94 (9.8)
Metformin + DPP-4 inhibitors	67 (7.0)
Metformin + sulfonylureas	25 (2.6)
Metformin + GLP-1 agonists	5 (0.5)
Sulfonylureas + DPP-4 inhibitors	5 (0.5)
Sulfonylureas + insulin	4 (0.4)
1 oral drug with insulin	117 (12.1)
Metformin + insulin + DPP-4 inhibitors	61 (6.3)
Metformin + sulfonylureas + DPP-4 inhibitors	29 (3.0)
Metformin + sulfonylureas + insulin	5 (0.5)
2 oral drugs with insulin	80 (8.3)
2 oral drugs without insulin	109 (11.3)
≥3 oral drugs with insulin	20 (2.1)
≥3 oral drugs without insulin	39 (4.0)
**TOTAL THERAPY (MONOTHERAPY AND COMBINATION)**	
Metformin	532 (55.2)
Insulin	413 (42.9)
DPP-IV inhibitors	294 (30.5)
Sulfonylureas	145 (15.0)
GLP-1 agonists^*^	35 (3.6)
Meglitinides	32 (3.3)
Glitazones	8 (0.8)
Patients aged >40 years + taking statin drug	672 (76.6)

* *GLP-1 agonists given only to obese patients with diabetes (BMI > 30 kg/m^2^)*.

### Factors Associated With Glycemic Control in Patients With Type 2 Diabetes

Table 3 presents the demographics and clinical characteristics of the study patients with diabetes divided into two groups (good glycemic control, HbA1c < 7%; poor glycemic control, HbA1c ≥ 7%). Among the demographics and clinical characteristics, only the levels of systolic blood pressure, total cholesterol, LDL cholesterol, HDL cholesterol, triglyceride, and fasting blood glucose had a significant association with glycemic control. Most clinical characteristics, except HDL cholesterol level, had a positive association with glycemic control; the patients with poor glycemic control (HbA1c level ≥ 7%) were likely to have higher systolic blood pressure and total cholesterol, LDL cholesterol, triglyceride, and fasting blood glucose levels. On the other hand, patients who had high HDL cholesterol levels were associated with good glycemic control (HbA1c level < 7%). Regarding comorbidities, neuropathy and foot ulcers were significantly associated with HbA1c levels; 79% of the patients with neuropathy and 85% of those with foot ulcers had poor glycemic control (HbA1c level ≥ 7%).

**Table 3:** Demographic and clinical characteristics distributed according to HbA1c level.

**Variable**	**HbA1c < 7%**	**HbA1c ≥ 7%**	***P-*value**
*Number of patients (%)*	284 (29.5%)	679 (70.5%)	
Age, mean ± SD (years)	52.8 ± 9.3	53.1 ± 9.6	0.59
*Sex, n (%)*			
*Male*	151 (53.2%)	332 (48.9%)	0.25
*Female*	133 (46.8%)	347 (51.1%)	
BMI, mean ± SD (kg/m^2^)	32.8 ± 7.1	33.3 ± 6.4	0.28
SBP, mean ± SD (mm/Hg)	132.2 ± 15.6	134.9 ± 16.3	0.035^*^
DBP, mean ± SD (mm/Hg)	71.7 ± 10.9	70.9 ± 11.1	0.32
TC, mean ± SD (mmol/L)	4.0 ± 0.9	4.2 ± 1.0	<0.001^*^
LDL-C, mean ± SD (mmol/L)	2.2 ± 0.8	2.3 ± 0.9	<0.01^*^
HDL, mean ± SD (mmol/L)	1.2 ± 0.4	1.1 ± 0.4	<0.01^*^
TG, mean ± SD (mmol/L))	1.3 ± 0.6	1.7 ± 1.1	<0.001^*^
FBS, mean ± SD (mmol/L)	7.0 ± 1.7	9.8 ± 3.5	<0.001^*^
Creatinine, mean ± SD	93.0 ± 70.9	88.9 ± 40.4	0.36
*Dyslipidemia, n (%)*	125 (27.8%)	324 (72.2%)	0.33
*Hypertension, n (%)*	115 (29.4%)	276 (70.6%)	1.0
*Nephropathy, n (%)*	103 (29.2%)	250 (70.8%)	0.93
*Neuropathy, n (%)*	72 (21.1%)	269 (78.9%)	<0.001^*^
*Retinopathy, n (%)*	54 (25.8%)	155 (74.2%)	0.22
*CHD, n (%)*	31 (32.6%)	64 (67.4%)	0.55
*Foot ulcer, n (%)*	8 (14.8%)	46 (85.2%)	<0.03^*^
*Stroke, n (%)*	5 (33.3%)	10 (66.7%)	0.78

* *Statistically significant (P < 0.05)*.

Table 4 presents the association between anti-diabetic medication and glycemic control in patients with type 2 diabetes. Two-thirds of the patients with diabetes with good glycemic control were significantly more likely to receive metformin as monotherapy or in combination. On the other hand, insulin use as monotherapy and total therapy was found to be significantly associated with poor glycemic control. Similarly, the use of DPP-4 inhibitors in total was associated with poor glycemic control. We further divided our patient sample based on insulin and non-insulin treatment to observe any association with glycemic control (Table 5). Patients treated with insulin had significantly higher fasting blood glucose, BMI, and HbA1c level as well as diabetes-related microvascular and macrovascular complications and had poorer glycemic control than those treated with oral anti-diabetic drugs.

**Table 4:** Association between anti-diabetic medication and glycemic control in T2D patients.

**Anti-diabetic medication**	**HbA1c < 7% (*n* = 284)**	**HbA1c ≥ 7% (*n* = 679)**	***P*-value**
**MONOTHERAPY**			
Metformin	102 (35.9%)	100 (14.7%)	<0.001^*^
Insulin	45 (15.8%)	151 (22.2%)	0.031^*^
DPP-IV inhibitors	17 (6.0%)	63 (9.3%)	0.118
Sulfonylureas	18 (6.3%)	45 (6.6%)	0.982
GLP-1 agonists	2 (0.7%)	14 (2.1%)	0.171
Meglitinides	1 (0.4%)	9 (1.3%)	0.296
Glitazones	0 (0%)	2 (0.3%)	1
1 oral drug without insulin	140 (49.3%)	233 (34.3%)	<0.001^*^
**COMBINATION THERAPY**			
Metformin + insulin	20 (7.0%)	74 (10.9%)	0.085
Metformin + DPP-4 inhibitors	24 (8.5%)	43 (6.3%)	0.299
Metformin + sulfonylureas	6 (2.1%)	19 (2.8%)	1
Metformin + GLP-1 agonists	4 (1.4%)	1 (0.1%)	0.028^*^
Sulfonylureas + DPP-4 Inhibitors	1 (0.4%)	4 (0.6%)	1
Sulfonylureas (SU) + insulin	1 (0.4%)	3 (0.4%)	1
1 oral drug with insulin	23 (8.1%)	94 (13.8%)	0.017^*^
Metformin + insulin + DPP-4 inhibitors	11 (3.9%)	50 (7.4%)	0.060
Metformin + SU + DPP-4 inhibitors	8 (2.8%)	21 (3.1%)	0.983
Metformin + sulfonylureas + insulin	1 (0.4%)	4 (0.6%)	1
2 oral drugs with insulin	14 (4.9%)	66 (9.7%)	0.019^*^
2 oral drugs without insulin	36 (12.7%)	73 (10.8%)	0.453
≥3 oral drugs with insulin	4 (1.4%)	16 (2.4%)	0.490
≥3 oral drugs without insulin	10 (3.5%)	29 (4.8%)	0.720
**TOTAL THERAPY**			
Metformin	184 (64.8%)	348 (51.3)	<0.001^*^
Insulin	86 (30.3%)	327 (48.2%)	<0.001^*^
DPP-IV inhibitors	69 (24.3%)	225 (33.1%)	0.008^*^
Sulfonylureas	38 (13.3%)	107 (15.8%)	0.400
GLP-1 agonists^*^	8 (2.8%)	27 (4.0%)	0.491
Meglitinides	6 (2.1%)	26 (3.8%)	0.245
Glitazones	0 (0%)	8 (1.2%)	0.113

* *Statistically significant (P < 0.05)*.

**Table 5:** Association between insulin treatment and glycemic control in T2D patients.

**Variable**	**Insulin treatment (*n* = 413)**	**Non-insulin treatment (*n* = 550)**	***P-*value**
Age, mean ± SD (years)	53.2 ± 9.8	52.8 ± 9.2	0.49
BMI, mean ± SD (kg/m^2^)	34.0 ± 7.1	32.5 ± 6.1	<0.01^*^
SBP, mean ± SD (mm/Hg)	135.5 ± 16.4	133.2 ± 15.8	0.05
DBP, mean ± SD (mm/Hg)	69.3 ± 11.9	72.4 ± 10.3	<0.001^*^
Total cholesterol, mean ± SD (mmol/L)	4.10 ± 1.1	4.2 ± 1.0	0.3
LDL, mean ± SD (mmol/L)	2.3 ± 0.9	2.3 ± 0.8	0.4
HDL, mean ± SD (mmol/L)	1.1 ± 0.4	1.2 ± 0.4	0.1
Triglycerides, mean ± SD (mmol/L)	1.7 ± 1.1	1.6 ± 1.0	0.3
FBG, mean ± SD (mmol/L)	9.4 ± 3.9	8.4 ± 2.8	<0.001^*^
HbA1c level, mean ± SD (%)	8.4 ± 1.7	7.7 ± 1.5	<0.001^*^
Patients with HbA1c <7%, n (%)	86 (20.8)	198 (36.0)	<0.001^*^
Macrovascular complications, n (%)	49 (11.9)	33 (6.0)	<0.03^*^
Microvascular complications, n (%)	284 (68.8)	317(57.6)	<0.001^*^

* *Statistically significant (p < 0.05). FBS, Fasting blood glucose. Diabetic macrovascular complications include coronary heart disease and stroke. Diabetic microvascular complications include nephropathy, retinopathy, neuropathy, and foot ulcers*.

### Adherence to Performance Indicators

Table 6 presents the level of adherence of patients with type 2 diabetes to performance indicators set by DDI and ADA. The proportion of patients with good glycemic control (Hb1AC level < 7%) significantly improved in the second year but became steady at 32.5% thereafter. Similarly, the proportion of patients with diabetes attaining optimal levels of LDL cholesterol (<2.6 mmol/L) and blood pressure (<140/90 mm/Hg) significantly increased over the first 3 years. In contrast, the proportion of patients with diabetes whose urine microalbumin or LDL cholesterol levels were measured at least once yearly decreased significantly after the first year and subsequently leveled off.

**Table 6:** Adherence of patients with type 2 diabetes to performance indicators by year.

**Variable**	**Year 1** ***N* = 881 (%)**	**Year 2** ***N* = 840 (%)**	**Year 3** ***N* = 771 (%)**	**Year 4** ***N* = 661 (%)**	***P*-value**
HbA1C measurement (≥1)	98.64	95.95	97.79	97.28	<0.10
LDL measurement (≥1)	94.89	88.21	89.49	88.35	<0.001
Urine microalbumin (≥1)	89.21	70.12	74.97	71.56	<0.001
HbA1C control (<9%)	65.36	79.28	77.32	77.60	<0.001
HbA1c control (<7%)	22.44	32.51	30.50	32.66	<0.01
Obese (BMI ≥ 30 kg/m^2^)	65.21	65.05	62.87	64.91	0.80
LDL-C level (<2.6 mmol/L)	43.03	55.83	62.99	63.14	<0.001
Blood pressure (<140/90 mmHg)	56.95	65.88	70.21	62.58	<0.001

## Discussion

This retrospective study was conducted to determine the level of metabolic control in patients with type 2 diabetes attending a specialist diabetes clinic in Kuwait and to investigate the factors that affect metabolic control. Our findings showed that most of the patients with diabetes (70.5%) did not attain the recommended target HbA1c level according to the ADA definition (<7%), with a mean HbA1c level of 8.5 ± 1.8%. This finding is in agreement with those of other studies conducted on patients with type 2 diabetes in several Gulf countries, whereby the prevalence of poor glycemic control ranged from 65 to 75% (12–14). In developed countries, several studies have reported that 35–67% of patients with type 2 diabetes have poor glycemic control (9, 10, 15–17).

It is recognized that tight glycemic control (HbA1c level < 7%) is necessary to reduce the risk of diabetes-related microvascular and macrovascular complications, as demonstrated by the UKPDS Group (7). Although the percentage of patients with HbA1c level of <7% improved dramatically after 1 year of attending our clinic (from 22.4 to 32.5%), it did not improve in the subsequent years. Despite the high obesity rates in our patients (65%), we observed no association between BMI and poor glycemic control. Further, several studies have showed the effect of weight on glycemic control (18, 19), but many studies have not observe this association (9, 20, 21). Another possible factor influencing poor glycemic control, which was not obtained in this study, was the duration of type 2 diabetes. Reportedly, patients with a type 2 diabetes duration of >10 years are likely to have a 15% higher HbA1c level than those with type 2 diabetes for a shorter duration (22).

Of the anti-diabetic drugs used by our patients with diabetes, metformin was most commonly prescribed and was used by >50% of the patients as monotherapy or in combination. Although our finding is in agreement with that of a previous study (23), a high proportion of patients have not been treated with metformin. In our study, the use of metformin as monotherapy or in combination was significantly associated with good glycemic control. This finding concurs with those of a systematic review of 35 double-blinded randomized controlled trials showing that metformin use as monotherapy, compared with placebo, was associated with an HbA1c reduction of 1.1% (24). The UKPDS Group has shown that metformin therapy for patients with type 2 diabetes reduced diabetic complications and death (7). Our data were not segmented based on diabetic complications, but our findings showed that patients treated with oral anti-diabetic drugs had fewer microvascular and macrovascular complications than those treated with insulin.

There is a high proportion of patients treated with insulin monotherapy, i.e., 20%, which is higher than that reported in previous studies (23, 25) and is not consistent with the ADA and European Association for the study of Diabetes (EASD) guidelines (26). Unlike metformin, insulin use as monotherapy or in combination with 1–2 oral anti-diabetic agents by our patients with diabetes was a predictor of poor glycemic control. Further stratification showed that insulin-treated patients had reduced probability of attaining glycemic targets of HbA1c <7% (21%) compared with those treated with oral anti-diabetic drugs (36%). Our findings are in agreement with those of some previous studies (9, 27–29), with one particular study demonstrating that insulin use is associated with an increase of 22.4% in HbA1c level relative to the use of diet or an oral anti-diabetic drug (22). Our findings indicate that a high proportion of patients with HbA1c >7% (~45%) are treated with monotherapy, highlighting the need to closely follow the ADA and EASD guidelines in the future. Although the deterioration in glycemic control is probably attributed to the progressive nature of diabetes, the choice of medications and their doses may also have important roles.

The clinical characteristics of patients with diabetes may also influence glycemic control, as suggested previously (12, 22). In our study, approximately 50% of the patients with diabetes had dyslipidemia as the most common comorbidity. Elevated lipid profile marker (LDL-C, total cholesterol, and triglycerides) levels were significantly and positively associated with poor glycemic control. According to Yurgin et al. (22), for every increase of 0.65 mmol/L in the total cholesterol level, the HbA1c value was higher by 2.6%. On the other hand, HDL cholesterol levels had a significant and positive influence on the improvement in HbA1c levels in our patients with diabetes. Hypertension was the second most common comorbidity in our patients (40%). This result is similar to those reported in studies conducted on patients with type 2 diabetes in a similar age group (17, 30, 31). According to these studies, the prevalence of hypertension increases to 60% by the age of 75 years. Similar to the effect of lipid marker levels, we observed a significant and positive association between systolic blood pressure and glycemic control. Our finding is in agreement with that of a large cross-sectional study on patients with type 2 diabetes conducted in Malaysia in which elevated blood pressure (≥130/80 mmHg) was found to be associated with poor glycemic control. In Singapore, a study on a large sample of patients with type 2 diabetes indicated that prehypertension levels are associated with poor glycemic control (32). It is recognized that intensive management of cholesterol and blood pressure is effective in preventing macrovascular disease in type 2 diabetes (6, 33).

As in other studies, the percentage of patients with microvascular complications in our study was higher than that of patients with macrovascular complications. Although nephropathy was the most common microvascular complication, only neuropathy and foot ulcers (manifestations of neuropathy) showed significant association with glycemic control. Compared with patients with diabetes with good glycemic control, those with poor glycemic control were 3–4-times more likely to have neuropathy and foot ulcers as microvascular complications, which is consistent with the findings of other studies (17, 18). In contrast, other investigators have shown that the presence of neuropathy did not significantly decrease the odds of achieving optimal glycemic control (34).

The ADA recommendations for blood pressure and LDL cholesterol levels were met by 62 and 63% of the patients, respectively, by follow-up year 4. Certainly, adherence to LDL cholesterol standards significantly improved in these patients in the past 4 years, which may indicate an aggressive lipid-lowering therapy approach. Nonetheless, only 5% of our patients met the triple targets for glycemia, blood pressure, and LDL cholesterol levels.

Our study has several limitations that are worth mentioning. First, the retrospective study design prevented us from determining a causal relationship between the clinical characteristics of the patients and HbA1c glycemic control. Second, we were not able to report the duration of diabetes for our patients because many patients had a late diagnosis and most of the patients were referred to our specialist diabetic center from primary care clinics with insufficient health data. Third, our study lacked data on physical activity and adherence to diet and lifestyle changes by our patients with diabetes, thus making it difficult to conduct a thorough assessment of diabetes management and the factors affecting glycemic control. Fourth, our study did not collect data on self-monitoring of blood glucose levels or detailed data on medicine dosage and adherence to treatment. Finally, glycemic control also depends on factors other than those assessed in this study, which were not assessed because they were beyond the scope of our study.

In conclusion, the results of this retrospective study indicate that the therapeutic management of type 2 diabetes in Kuwait is suboptimal. Therapeutic strategies should ensure better adherence to ADA guidelines and evaluate high obesity rates and any lifestyle changes followed by patients. Emphasis on diabetes education and self-empowerment is the key to successful management of this disease. Further longitudinal studies are warranted to observe the trends of diabetes and its glycemic control and the associated short- and long-term complications.

## Ethics Statement

This study was carried out after ethical approval from the Ethical Review Committee at DDI in 2014 to allow access to the patient data from the KBHR electronic health record database. All patients attending the clinic gave a written informed consent in accordance with the Declaration of Helsinki.

## Author Contributions

YA-K contributed to conception and design of the study. DB and YA-K facilitated regulatory approval for this study and data retrieval from the KBHR as well as overall organization of the study. AC conducted statistical analysis with the assistance of MQ. MQ drafted the first version of the manuscript. MQ and JT contributed to the revision of the manuscript. All authors contributed and approved the final version of the manuscript.

### Conflict of Interest Statement

The authors declare that the research was conducted in the absence of any commercial or financial relationships that could be construed as a potential conflict of interest.
